# Multifunctional titanium dioxide nanoparticles biofabricated via phytosynthetic route using extracts of *Cola nitida*: antimicrobial, dye degradation, antioxidant and anticoagulant activities

**DOI:** 10.1016/j.heliyon.2020.e04610

**Published:** 2020-08-04

**Authors:** P.O. Akinola, A. Lateef, T.B. Asafa, L.S. Beukes, A.S. Hakeem, H.M. Irshad

**Affiliations:** a Laboratory of Industrial Microbiology and Nanobiotechnology, Department of Pure and Applied Biology, Ladoke Akintola University of Technology, PMB 4000, Ogbomoso, Nigeria; b Nanotechnology Research Group (*NANO*^*+*^), Ladoke Akintola University of Technology, PMB 4000, Ogbomoso, Nigeria; c Department of Mechanical Engineering, Ladoke Akintola University of Technology, PMB 4000, Ogbomoso, Nigeria; d Microscopy and Microanalysis Unit, School of Life Sciences, University of KwaZulu-Natal, Private Bag X01, Scottsville, PieterMaritzburg 3209, South Africa; e Center for Excellence in Nanotechnology, King Fahd University of Petroleum and Minerals, Saudi Arabia; f Faculty of Materials and Chemical Engineering, Ghulam Ishaq Khan Institute of Engineering, Science and Technology, Pakistan

**Keywords:** Materials science, Materials analysis, Nanotechnology, Microbiology, Biomedical engineering, *Cola nitida*, Phytosynthesis, Titanium dioxide (TiO_2_) nanoparticles, Biomedical applications

## Abstract

First study of phytosynthesis of TiO_2_ NPs using the leaf (KL), pod (KP), seed (KS) and seed shell (KSS) extracts of kola nut tree (*Cola nitida*) is herein reported. The TiO_2_ NPs were characterized and evaluated for their antimicrobial, dye degradation, antioxidant and anticoagulant activities. The nearly spherical-shaped particles had λmax of 272.5–275.0 nm with size range of 25.00–191.41 nm. FTIR analysis displayed prominent peaks at 3446.79, 1639.49 and 1382.96 cm^−1^, indicating the involvement of phenolic compounds and proteins in the phytosynthesis of TiO_2_ NPs. Both SAED and XRD showed bioformation of crystalline anatase TiO_2_ NPs which inhibited multidrug-drug resistant bacteria and toxigenic fungi. The catalytic activities of the particles were profound, with degradation of malachite green by 83.48–86.28 % without exposure to UV-irradiation, scavenging of DPPH and H_2_O_2_by 51.19–60.08 %, and 78.45–99.23 % respectively. The particles as well prevented the coagulation of human blood. In addition to the antimicrobial and dye-degrading activities, we report for the first time the H_2_O_2_ scavenging and anticoagulant activities of TiO_2_ NPs, showing that the particles can be useful for catalytic and biomedical applications.

## 1 Introduction

Nanotechnology is a vast field that is making impacts in all fields of human life. Nanotechnology is the manufacturing and exploitation of materials whose components exist at the nanoscale (1–100 nm in size). Nanotechnology explores electrical, optical and magnetic activities as well as structural behaviour at the molecular and sub-molecular level making them suitable for wide range of applications including biomedicine [1, 2]. Nanotechnology is not only concerned about the size of very small things; it is the revolutionary science and the art of controlling matter at the atomic or molecular scale to produce products with some desired and novel features or properties. When a matter is as small as 1–100 nm, many of its features will change easily with a number of unique features that are different from the bulk form.

Among several metal oxide nanoparticles, titanium dioxide nanoparticles (TiO_2_ NPs) are non-toxic with oxidation potency and elevated stability to light resulting into their broad applications in environmental remediation [3, 4]. In addition, TiO_2_ NPs possess fascinating dielectric, optical, antimicrobial, chemical and catalytic properties which lead to industrial applications such as cosmetics, pigment, fillers, whitening and brightening of foods, in personal care products like toothpaste, and photocatalyst [5, 6, 7, 8]. Furthermore, its low toxicity and biocompatibility have expanded the applications in food and biomedical areas as bone tissue engineering, dentistry and drug manufacturing [9, 10, 11, 12]. In view of the important applications of TiO_2_ NPs, it has been efficiently synthesized using biological resources such as bacteria, fungi and plants in eco-friendly and simple way [13, 14]. To further broaden the horizon of synthesis and applications of nanoparticles, researchers continue to explore different bioresources for their production [15, 16].

*Cola nitida* (Sterculiaceae) is an evergreen tree which grows to a height of 12–20 m, and is commonly found in Nigeria, Ghana, Sierra Leone, Ivory Coast and Liberia. The trunk can measure up to 1.5 m in diameter along with older trees which develop buttresses. The bark is thick and fibrous, with deep longitudinal fissures. It is grey or brownish-grey, with pinkish-red wood which becomes visible when the bark is damaged. It has been cultivated in other parts of the World such as India, Australia, Malaysia, Trinidad, Jamaica, Brazil, and Hawaii [17]. Kola nut tree contains compounds that have antimicrobial [18, 19], anti-inflammatory [20], antidiuretic [21], antidiabetic [22], antioxidative [23] and anticancer [24] activities. The plant has also been used to treat cardiovascular disease, whooping cough and asthma [25, 26].

Aside the pharmaceutical potentials of *C. nitida,* different parts of the plant have been employed as microbial substrate to produce enzyme and enhancement of nutritional qualities [27, 28] and also for the synthesis of silver and silver-gold alloy nanoparticles [29, 30, 31, 32, 33, 34, 35] for different biomedical and environmental applications. This work therefore seeks to extend the border of the potential applications of extracts of different parts of kola nut in nanobiotechnology. Evidently, this represents the first study on synthesis of TiO_2_ nanoparticles using extracts of *C. nitida* for biomedical and catalytic applications. Suffice also, to state that until now, there is no report on the H_2_O_2_ scavenging and anticoagulant properties of TiO_2_ NPs.

## 2 Materials and methods

### 2.1 Sample collection and preparation

Leaves and fruits of *C. nitida* were obtained from a local farm in Ogbomoso, Oyo State. The seeds and seed shells were removed from the pods and these were cut into smaller pieces. Thereafter, chopped seeds, seed shells, pods and leaves were air-dried for 5 days under ambient condition, after which they were milled separately into powder using an electric blender and stored in airtight container [31]. To obtain the extracts, one gram of each sample was dispersed in 100 ml of water, heated at 60 °C for 1 h and clarified using Whatman No. 1 filter paper followed with centrifugation at 4000 rpm for 10 min.

### 2.2 Phytosynthesis and characterization of TiO_2_ NPs

Prior to phytosynthesis, the precursor was obtained by preparing 1 mM of TiO(OH)_2_ (Sigma-Aldrich, USA) in distilled water. The particles (KP-, KL-, KS- and KSS TiO_2_ NPs) were prepared as follows: 20 ml of each extract was added distinctively to a reaction vessel containing 100 ml of 1 mM TiO(OH)_2_ at room temperature for 1 h to observe the colour change. The precursor TiO(OH)_2_ served as the control. The nanoparticles were characterized using different analytical techniques as earlier described [31, 36]. UV-vis spectroscopy was investigated by scanning from 190 to 900 nm on a spectrophotometer (Cecil, USA), while FTIR spectra were obtained on Affinity-1S spectrometer (Shimadzu, UK), after dried particles were mixed with KBr pellets. TEM images were obtained by placing a drop of colloidal TiO_2_ NPs separately on a 200 mesh hexagonal copper grid (3.05 mm) (Agar Scientific, Essex, UK) coated with 0.3 % formvar dissolved in chloroform and examined on JEM-1400 TEM (JEOL, USA). Furthermore, micrographs and elemental compositions of the colloidal particles were obtained on a LYRA 3 TESCAN FESEM coupled with Energy dispersive X-ray (EDX) (Oxford), that was operated at 20.0 kV. Also, XRD was used to analyze the nanoparticles.

### 2.3 Selection of antibiotic resistant bacterial isolates

Test bacterial isolates from clinical investigations were obtained from LAUTECH Teaching Hospital, Ogbomoso and screened for susceptibility using a panel of antibiotics on Mueller Hinton Agar plates by disc diffusion assay as previously demonstrated [37]. Gram positive discs (Rapid Labs., UK) impregnated with antibiotics containing (μg): ceftazidime (Caz), 30; cefuroxime (Crx), 30; gentamicin (Gen), 10; cefixime (Cxm), 5; ofloxacin (Ofl), 5; augmentin (Aug), 30; nitrofurantoin (Nit), 300; and ciprofloxacin (Cpr), 5, as well as Gram negative discs containing (μg): ceftazidime (Caz), 30; cefuroxime (Crx), 30; gentamicin (Gen), 10; ciprofloxacin (Cpr), 5; ofloxacin, (Ofl), 5; amoxycillin (Aug), 30; nitrofurantoin (Nit), 300; and ampicillin (Amp), 10 were used for the evaluation. The plates were incubated at 37 °C for 48 h, and afterwards, the zones of inhibition were examined and interpreted [38]. The multi-drug resistant isolates that included *Staphylococcus aureus* obtained from pus, *Pseudomonas aeruginosa* obtained from wound, *Escherichia coli* and *Klebsiella pneumoniae* obtained from urine were selected for further investigation.

### 2.4 Antibacterial and antifungal activities of synthesized TiO_2_ NPs

The antibacterial efficacy of the phytosynthesized TiO_2_ NPs was investigated separately against strains of clinical bacterial isolates using the modified broth culture method as described [39]. Eight milliliter of 24 h-old cultures of bacterial isolates containing 1.0 × 10^6^ cfu/ml were exposed to 1 ml of respective nanoparticles in the concentration range of 20–80 μg/ml and incubated at 37 °C for 24 h. Bacterial suspension without exposure to the nanoparticles serve as the control. The growth of the bacterial isolates was measured at 600 nm using UV-visible spectrophotometer. The percentage growth inhibition was estimated using Eq. (1):

(1) $$\text{Percentage}\;\text{bacterial}\;\text{growth}\;\text{inhibition}=\frac{A\;control-A\;test}{A\;control}\times 100\%$$

where A is the absorbance.

The antifungal activities of KL-TiO_2_ NPs, KP-TiO_2_ NPs, KS-TiO_2_ NPs and KSS-TiO_2_ NPs were determined using mycelial growth inhibition test [40] by inoculating 7 mm disc of 48 h-old culture of *Aspergillus niger* and *Fusarium solani* on potato dextrose agar that have been incorporated with TiO_2_ NPs at final concentrations of 60 and 80 μg/ml. The control plate contained no nanoparticles. All the plates were incubated at 28 ± 2 °C for 72 h. The radial fungal growths in all the plates were measured and the percentage growth inhibitions were calculated using Eq. (2):

(2) $$\text{Percentage}\;\text{fungal}\;\text{growth}\;\text{inhibition}=\frac{D\;control-D\;test}{D\;control}\times 100\%$$

where D is the diameter of fungal growth.

### 2.5 Catalytic activity of TiO_2_ NPs

The dye degrading abilities of the phytosynthesized KL-TiO_2_ NPs, KP-TiO_2_ NPs, KS-TiO_2_ NPs and KSS-TiO_2_ NPs were investigated separately using malachite green according to Lateef *et al.* [41] under ambient light in the laboratory. In this case, 1 ml of nanoparticles (10, 20, 40 and 80 μg/ml) was reacted with 9 ml of malachite green (40 ppm), while the control was without exposure to the nanoparticles. The reaction took place for 24 h at room temperature on rotary shaker (100 rpm), after which the absorbance readings were obtained at 619 nm. Percentage dye degradation was calculated using Eq. (3) [42].

(3) $$\text{Percentage}\;\text{dye}\;\text{degradation}=\frac{A\;control-A\;test}{A\;control}\times 100\%$$

where A is the absorbance value.

### 2.6 Antioxidant activities of TiO_2_ NPs

#### 2.6.1 DPPH radical-scavenging activity

The modified methods of Azeez *et al.* [43] and Lateef *et al.* [44] were used to study the free radical-scavenging activity of the KL-TiO_2_ NPs, KP-TiO_2_ NPs, KS-TiO_2_ NPs and KSS-TiO_2_ NPs using 2,2-diphenyl-1-picrylhydrazyl or DPPH (Sigma-Aldrich, Germany). About 1 ml of graded concentration of the TiO_2_ NPs was added separately to 4.0 ml of a methanolic solution of 0.1 mM DPPH. The mixture was mixed and allowed to react for 30 min at room temperature, after which absorbance readings were taken at 517 nm. The blank was 0.1 mM methanol DPPH which served as control. The scavenging percentage of DPPH was calculated according to Eq. (4).

(4) $$\text{Percentage}\;\text{DPPH}\;\text{scavenging}\;\text{effect}\;=\frac{A\;blank-A\;sample}{A\;blank}\times 100\%$$

#### 2.6.2 Hydrogen peroxide scavenging activity

The ability of the phytosynthesized TiO_2_ NPs to scavenge hydrogen peroxide was determined according to the methods of Bhakya *et al.* [45]. Hydrogen peroxide (40 mM) was prepared in phosphate buffer (pH 7.4), and 0.6 ml of it was reacted with 4 ml of each of the TiO_2_ NPs at room temperature for 20 min. The H_2_O_2_ solution was used as the control while distilled water was used as blank and the absorbance readings was read at 610 nm. The percentage peroxide scavenging activity was calculated using Eq. (5):

(5) $$\text{\%}\;\text{scavenging}\;\text{of}\;{\text{H}}_{2}{\text{O}}_{2}=\frac{A\;control-A\;sample}{A\;control}\times 100$$

where A is the absorbance.

### 2.7 Anticoagulant activity of TiO_2_ NPs

The anticoagulant activity of the TiO_2_ NPswas investigated separately by mixing 0.5 ml of a donor's blood with 1 ml of 80 μg/ml of TiO_2_ NPs. The control samples was set up using EDTA bottle, TiO(OH)_2_ solution and extracts of kola leaf (KLE), pod (KPE), seed (KSE), and seed shell (KSSE). The mixtures were held at room temperature for 1 h and then examined for coagulation of blood [46].

## 3 Results and discussion

### 3.1 Phytosynthesis of TiO_2_ NPs

**Figure 1:**
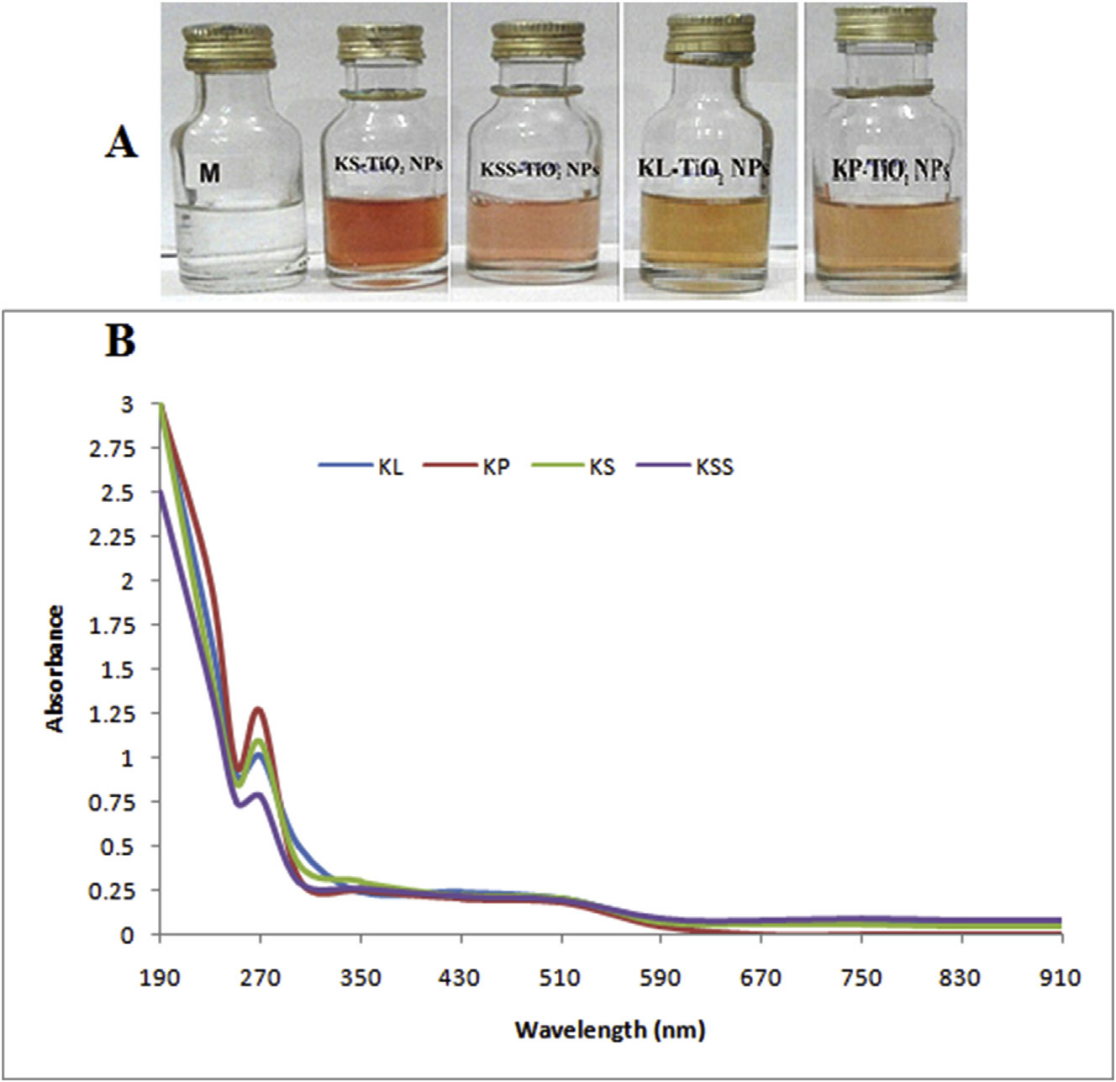
(a) Colour change in the synthesis of colloidal TiO_2_ NPs (b) UV-vis spectra of colloidal solution of TiO_2_ NPs.

The TiO_2_ NPs were synthesized by a novel, simple, and green biological procedure using pod, seed, seed shell and leaf extracts of *C. nitida*. The TiO(OH)_2_ solution lacking aqueous extract was observed to show no colour change, and there was no evidence for the formation of nanoparticles (Figure 1a). But there was a noticeable colour change to golden yellow after the separate addition of extracts due to the reduction of titanium ions. Several authors have reported different colours of TiO_2_ NPs colloidal solution like dark brown by Ganesan *et al.* [47] using extract of *Ageratina altissima* (L.), while Dobrucka [48] reported green colour using aqueous extract of *Echnacea pupurea* herba. Nithya *et al.* [49] and Rajakumar *et al.* [50] both reported light green colloidal TiO_2_ NPs using the leaf extract of *Aloe vera* and *Eclipta prostrata* respectively.

### 3.2 Characterization of biosynthesized TiO_2_ NPs

UV-Vis absorption spectroscopy is significant in monitoring the formation and stability of metal nanoparticles in aqueous solution. The spectrum of the metal nanoparticles is due to a lot of factor which include the size of particle, shape and agglomeration (particle-particle interaction) with the medium. Figure 1b revealed the UV-vis absorption spectra of the nanoparticles within the range of 272.5–275 nm. These values are similar to 270 nm obtained by Valli and Jayalaskshmi [51] using *Erythrina variegata* leaf extract for the synthesis of TiO_2_ NPs. Also, Dobrucka [48] reported maximum absorbance at 280 nm for TiO_2_ NPs using *Echinacea pupurea* herba.

**Figure 2:**
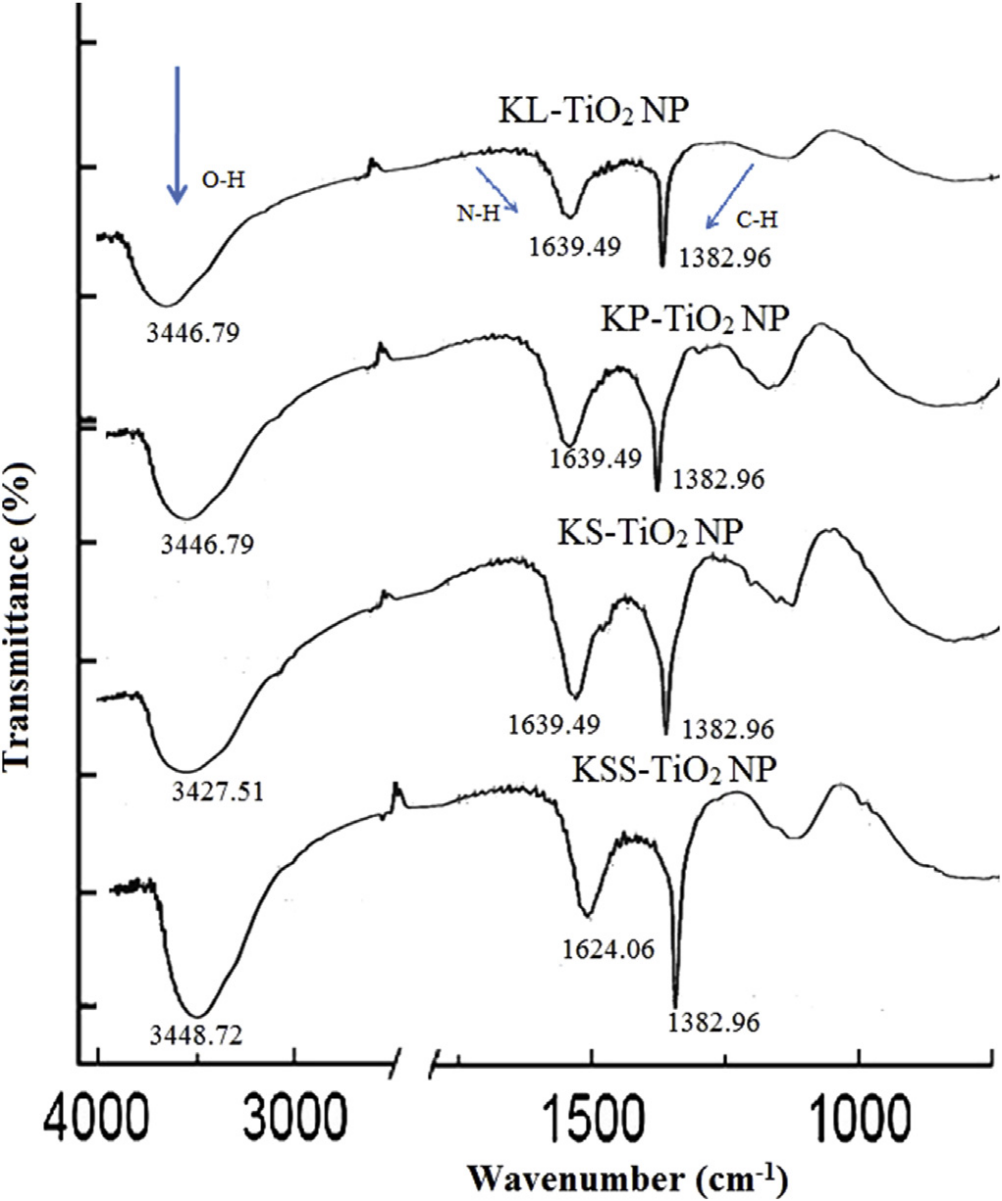
The FTIR spectra of phytosynthesized TiO_2_ NPs.

The FTIR analysis was used to identify the capping, reducing as well as stabilizing capacity of the extracts. It was used to determine the functional groups that are separately attached to TiO_2_ NPs. The FTIR spectra showed the presence of three prominent peaks in the nanoparticles (Figure 2). The stretches 3427–3448 and 1624-1639 cm^−1^ correspond to O–H stretch of carboxylic acid or N–H of amines respectively which shows that phenolics and protein are involved in the biosynthesis of TiO_2_ NPs, while 1382.96 cm^−1^ corresponds to C–H in plane bend stretching of alkenes [52]. This proves that TiO_2_ NPs were synthesized with *C. nitida* compounds involved in the biological reduction of TiO(OH)_2_ and subsequent capping of the synthesized TiO_2_ NPs. Kola nut as well as its parts have been reported to have approximately 15.24% protein, with sufficient abundance of kolatine, alkaloids, phenolic compounds, essential oils, caffeine, theobromine and nicotine [19, 53, 54].

**Figure 3:**
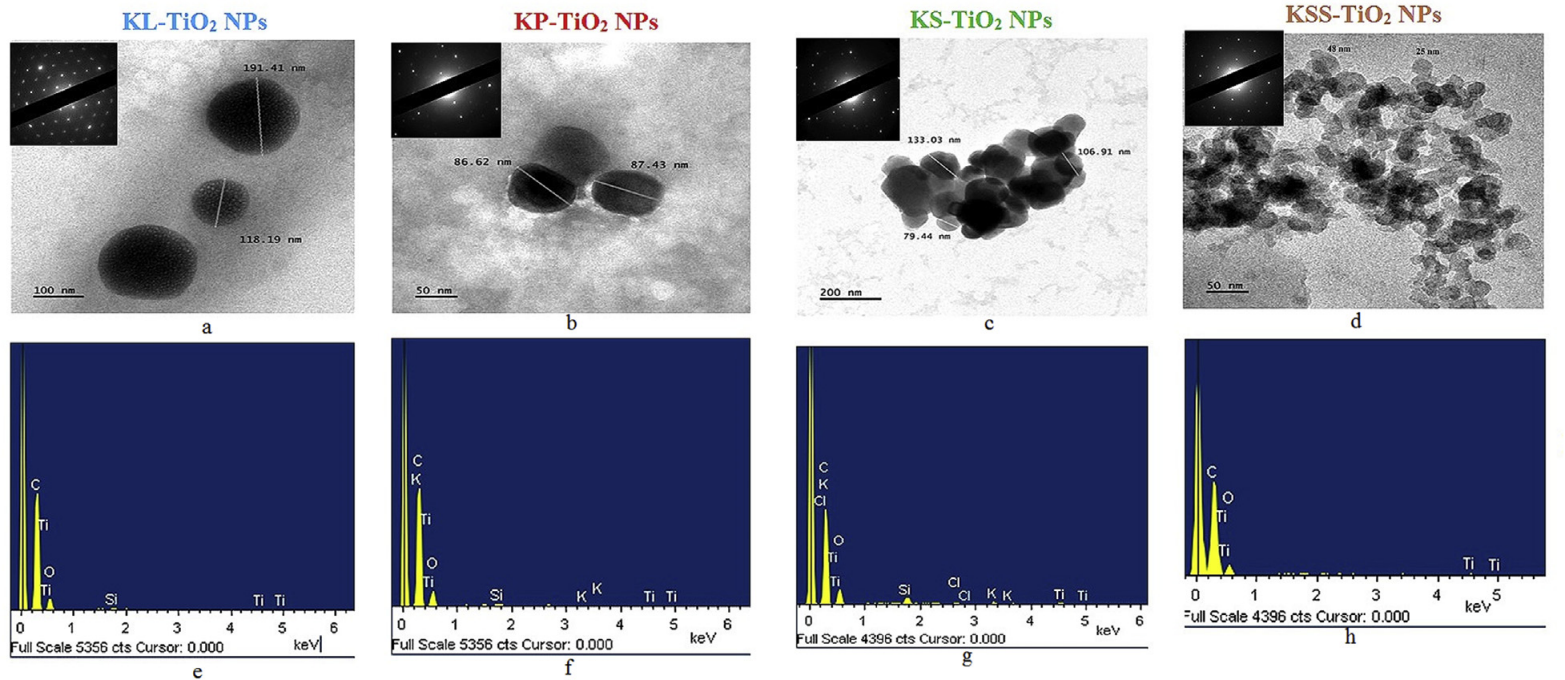
(a–d) Transmission electron micrographs (inset, selected area electron diffraction pattern) and (e–h) energy dispersive x-ray signals of phytosynthesized TiO_2_ NPs.

TEM images as shown in Figures 3a–d revealed that the TiO_2_ NPs were polydispersed with sizes in the range of 118–191.41, 86.62–87.43, 79.44–133 and 25–50 nm for KL-TiO_2_ NPs, KP-TiO_2_ NPs, KS-TiO_2_ NPs and KSS-TiO_2_ NPs respectively and were of near spherical morphology. The SAED of the biosynthesized TiO_2_ NPs as shown in the inset of Figures 3a-d yielded ring patterns. This demonstrates that the samples are made up of crystalline particles. Some scattered bright spots seen in the diffraction patterns indicate slightly larger crystalline grain size. These observations are similar to earlier reports on TiO_2_ NPs [55, 56]. EDX analysis showed the titanium peaks within 0.4–4.9 keV (Figure 3e-h) as previously observed [57, 58]. Other elements such C, Si, Cl and K that are depicted in EDX are impurities from the plant extracts.

**Figure 4:**
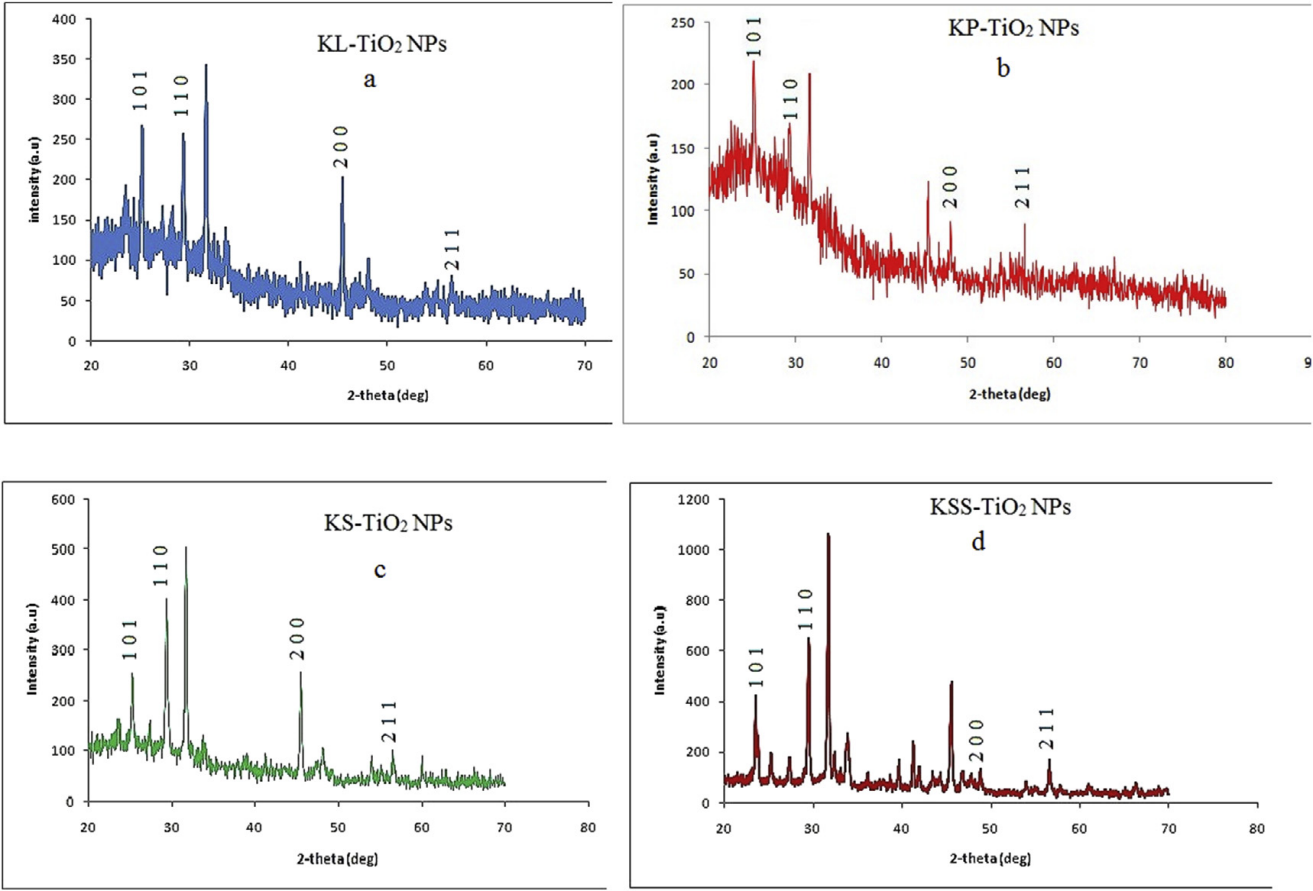
X-ray diffraction patterns of phytosynthesized TiO_2_ NPs (a, KL; b, KP; c, KS; d, KSS).

The XRD patterns of phytosynthesized TiO_2_ NPs (Figures 4a–d) showed major peaks appeared with 2θ values around 25.0°, 29.0°, 47.0° and 56. 0˚depicting the formation of anatase TiO_2_ NPs as reported by previous authors [51, 57, 58] and indexed to JCPDS file no. 84–1285 [59]. Using the Scherrer's equation

(6) $$\text{D}=\frac{\text{K}ʎ}{\text{β ​cosθ}}\text{,}$$

The average sizes of the particles were 143.01, 85.16, 85.81 and 34.34 nm for KL-, KP-, KS- and KSS- TiO_2_ NPs, respectively. The unidentified peaks are ascribed to impurities on the particles as earlier evidenced in the EDX spectra.

### 3.3 Antimicrobial activities of phytosynthesized TiO_2_ NPs

**Figure 5:**
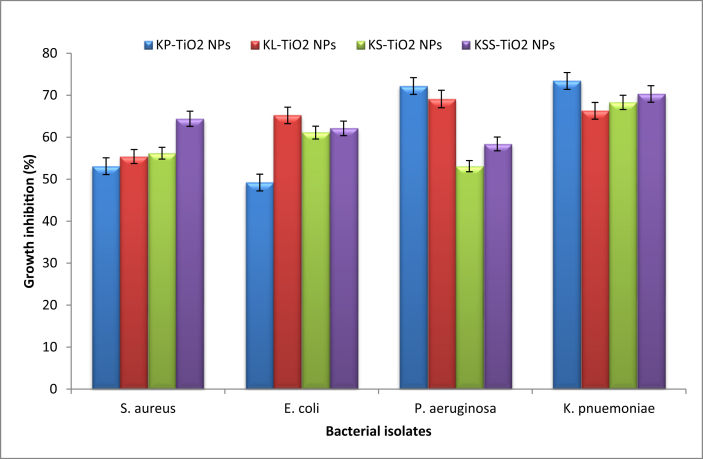
Antibacterial activities of TiO_2_ NPs at 80 μg/ml using broth method.

The susceptibility test of the bacterial isolates showed that *S. aureus* (pus) was resistant to crx, caz, aug, cxc, ery; *P. aeruginosa* (wound) was resistant to amp, caz, crx, gen, cpr, ofl, aug; while *E. coli* (urine) and *K. pneumoniae* (wound) were resistant to aug, amp, caz and crx. However, the isolates were sensitive to the TiO_2_ NPs, while they were not inhibited by the plant extracts and the precursor. The highest percentage growth inhibition of the synthesized TiO_2_ NPs at 80 μg/ml ranged from 49.2% against *E. coli* to 73.4% against *K. pneumoniae* by KP-TiO_2_ NPs (Figure 5). The cumulative growth inhibitions by the particles were 59.68, 63.80, 61.98 and 64.00% for KS-TiO_2_ NPs, KSS-TiO_2_ NPs, KP-TiO_2_ NPs and KL-TiO_2_ NPs respectively. Among the isolates, *K. pneumoniae* was the most sensitive, with the average growth inhibition of 69.58% by the TiO_2_ NPs, while *S. aureus* had the least growth inhibition of 57.28%. Different types of biosynthesized TiO_2_ NPs have shown the ability to inhibit the growth of antibiotic susceptible strains of *S. aureus, E. coli, P. aeruginosa* and *K. pneumoniae* [56, 58, 60, 61, 62] at concentrations of 20–250 μg/ml. It is noteworthy in the present investigation that the phytosynthesized TiO_2_ NPs inhibited growth of bacteria that showed drug resistance to 4–7 antibiotics. This is an indication that the synthesized TiO_2_ NPs can be useful to combat drug resistance among bacteria in clinical and environmental applications. Evidences have shown that TiO_2_ NPs can inhibit or kill bacterial cells through adherence to cell wall to initiate damage and leakage of intracellular contents, release of Ti^4+^, generation of reactive oxygen species and hydroxyl radicals [57, 58, 61].

**Figure 6:**
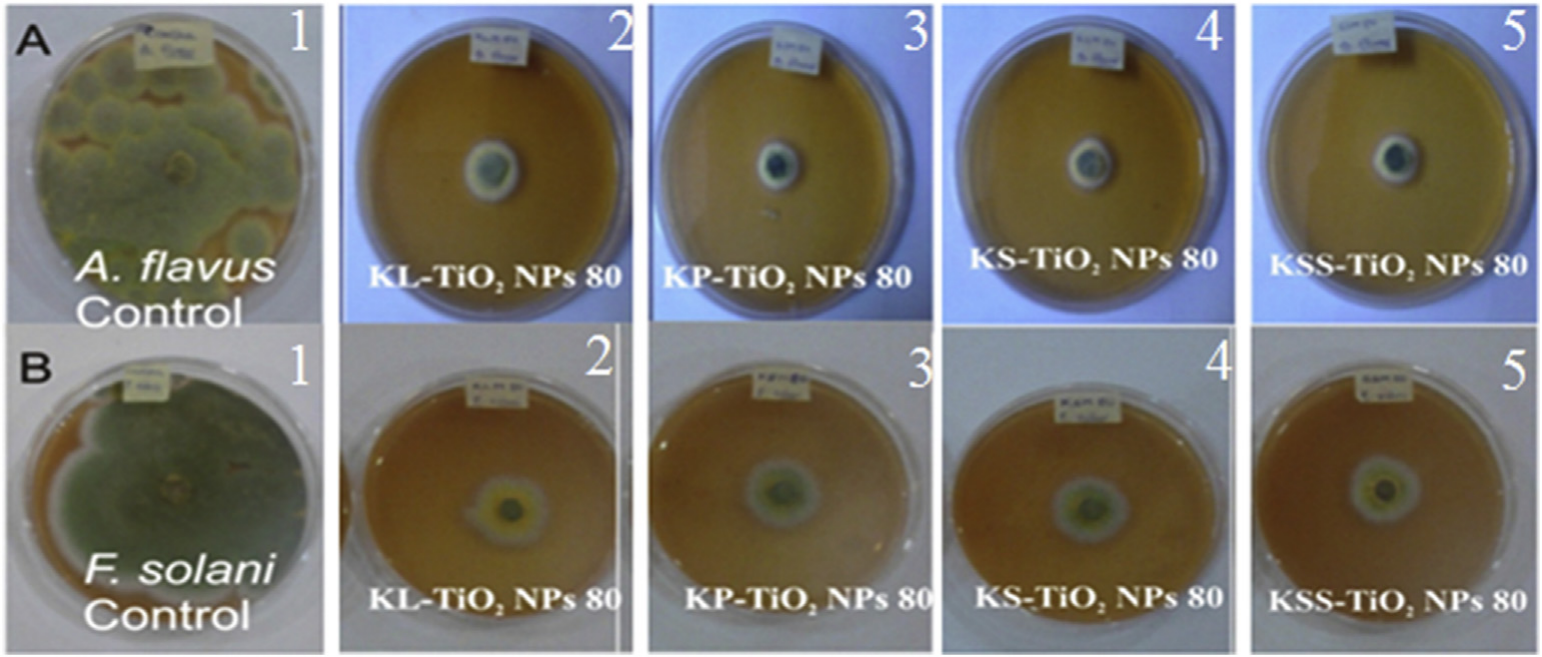
Antifungal activities of phytosynthesized TiO_2_ NPs at 80 μg/ml against (a) *A. flavus* (b) *F. solani* (1, control; 2, KL; 3, KP; 4, KS; 5, KSS).

The biosynthesized TiO_2_ NPs demonstrated significant antifungal activities of 71.44–84.72% revealing differences among the treatments but in dose-dependent manner. At 80 μg/ml, KL-TiO_2_ NPs, KP-TiO_2_ NPs, KS-TiO_2_ NPs and KSS-TiO_2_ NPs had growth inhibition of 82.13, 84.72, 84.41 and 84.53% respectively against *Aspergillus flavus,* while inhibition of 79.32, 76.16, 76.13 and 79.22% were obtained against *Fusarium solani* (Figure 6). Growth inhibition of *A. niger* by biosynthesized TiO_2_ NPs has been reported in literature [52, 58], however there is dearth of data on the activities of green synthesized TiO_2_ NPs against *A. flavus* and *F. solani,* which are important toxigenic fungi in agricultural and food production. Thus, these kola nut-mediated TiO_2_ NPs can find useful application in preventing growth of the toxigenic fungi in food production.

### 3.4 Catalytic activity of phytosynthesized TiO_2_ NPs

**Figure 7:**
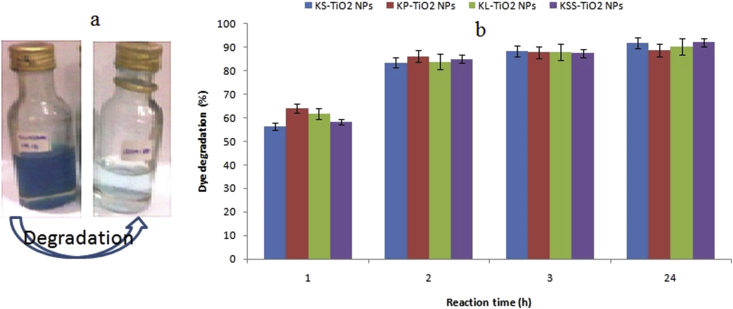
Degradation of malachite green by phytosynthesized TiO_2_ NPs (a, visual observation; b, % dye degradation).

Malachite green was effectively degraded by phytosynthesized TiO_2_ NPs at different concentrations of 10, 20, 40, 60, and 80 μg/ml. However, best performances were obtained at 80 μg/ml in the range of 56.42–92.12% within a period of 24 h (Figure 7) without UV-irradiation. At 2 h of reaction, degradations of malachite green by 83.48–86.28% were achieved by the nanoparticles. In all, the final dye degrading activities of the particles are comparable. Both green and chemically-synthesized TiO_2_ NPs have been used to degrade malachite green [63, 64, 65, 66] usually under photocatalytic condition. Therefore, the phytosynthesized TiO_2_ NPs used in this study can be used to remediate malachite green polluted effluent in the textile industry. Nanoparticles have been shown to serve as electron transfer mediators between the biomolecules on the surface of particles and dye, thereby catalyzing the degradation of the dye through redox reaction [46, 67].

### 3.5 Antioxidant activities of phytosynthesized TiO_2_ NPs

#### 3.5.1 DPPH radical-scavenging activity of TiO_2_ NPs

**Figure 8:**
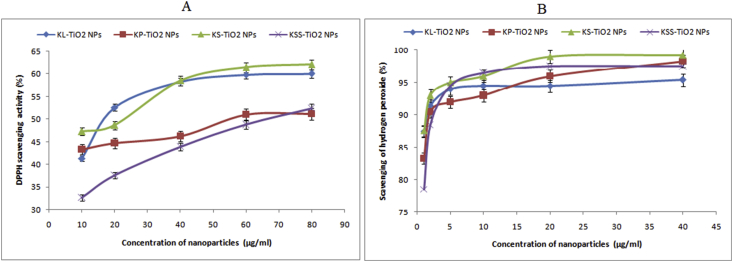
Scavenging activities of phytosynthesized TiO_2_ NPs (a) DPPH and (b) H_2_O_2_.

The biosynthesized TiO_2_ NPs were capable of causing decolourization of DPPH after the incubation time and thus indicated that they are antioxidant in nature. DPPH was scavenged at tested concentrations of 10–80 μg/ml in dose dependent manner to yield responses of 32.61–62.06% (Figure 8a). Among the phytosynthesized TiO_2_ NPs, kola seed mediated particles were the most potent, while the least activities were obtained for KSS-TiO_2_ NPs having highest performance of 52.37%. Authors have reported DPPH scavenging activities of 2–85% for TiO_2_ NPs biosynthesized using plants such as *Psidium guajava, Pithecellobium dulce, Lagenaria siceraria, Allium eriophyllum* and *Artemisia haussknechtii* [60, 68, 69, 70] at concentrations ranging from 1-1000 μg/ml. The present report proves that TiO_2_ NPs produced using kola extracts can attack disease-causing free radicals.

#### 3.5.2 Hydrogen peroxide scavenging activities of phytosynthesized TiO_2_ NPs

The phytosynthesized TiO_2_ NPs showed high significant scavenging activities of 78.45–99.23 % (Figure 8b) against H_2_O_2_ in dose dependent manner. At the moment, there are no reports on the H_2_O_2_ scavenging activity of TiO_2_ NPs, however authors have reported the scavenging activities of cerium oxide, silver, gold, silver-gold alloy and platinum nanoparticles on H_2_O_2_ [36,40,45,71,72] with very high performances. Thus, this study represents the first reference on hydrogen scavenging activity by TiO_2_ NPs, which can be explored in the environmental degradation of H_2_O_2_ to prevent the generation of highly reactive and hazardous hydroxyl radicals.

### 3.6 Anticoagulant activities of phytosynthesized TiO_2_ NPs

**Figure 9:**
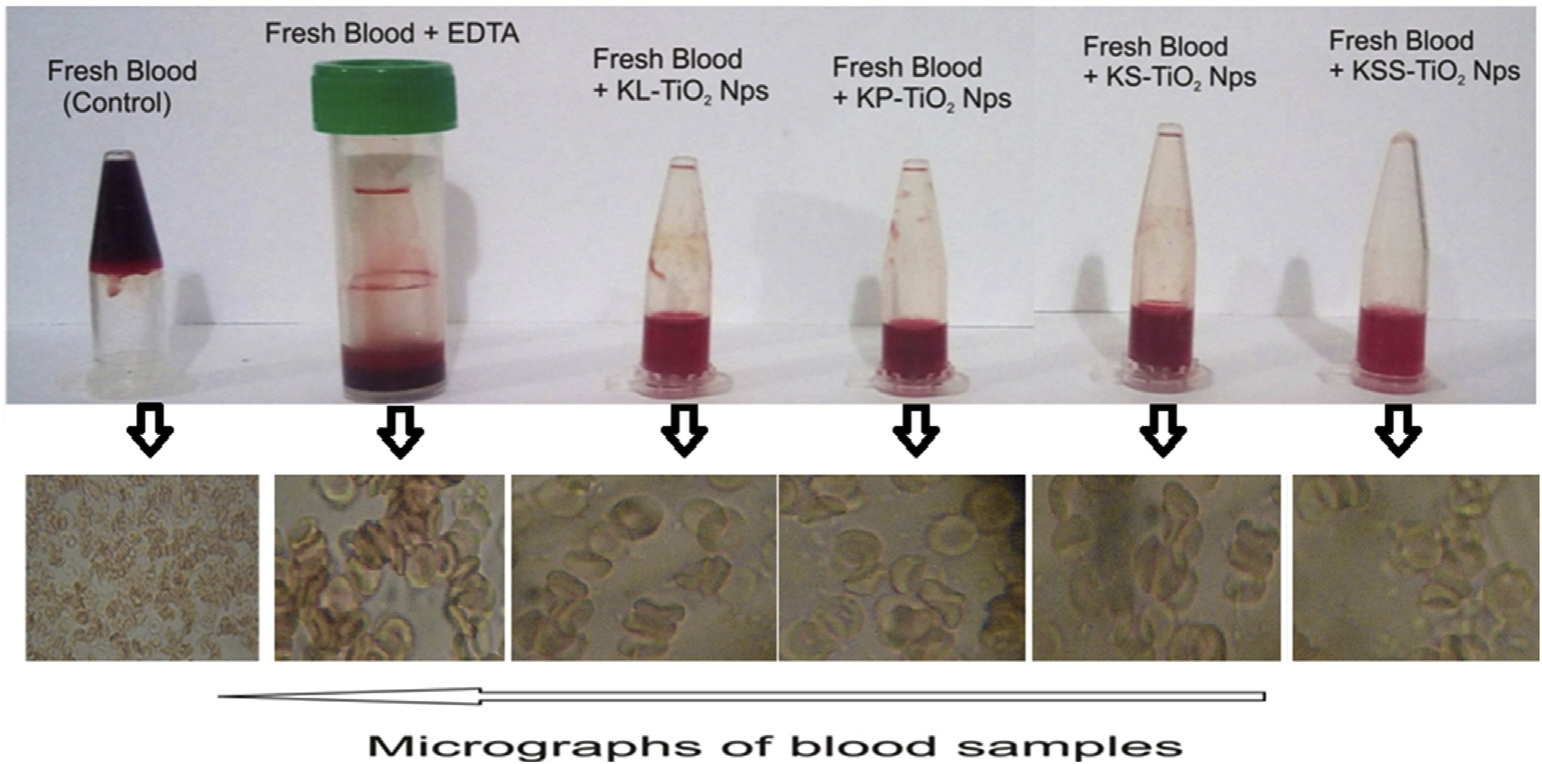
Anticoagulant activities of TiO_2_ NPs synthesized using extracts of different parts of *Cola nitida* on human blood.

The coagulation of human blood *in vitro* was prevented by all the TiO_2_ NPs retaining the morphology of red blood cells as obtained in the fresh blood collected in the EDTA bottle (Figure 9). This was similar to our earlier reports on the anticoagulant activities of silver, gold and silver-gold alloy nanoparticles [40, 73, 74, 75]. The extracts as well as TiO(OH)_2_ were not active as anticoagulants leading to failure to prevent coagulation of blood. Though there is growing trend in the anticoagulant activities of nanoparticles [73, 76, 77] for improved management of blood coagulation disorders, there is no report on the anticoagulant activities of TiO_2_ NPs until now.

## 4 Conclusion

This study has shown the effectiveness of leaf, pod, seed and seed shell extracts of *C. nitida* for the synthesis of TiO_2_ NPs. The particles were of near spherical morphology, crystalline and anatase in nature with maximum UV absorbance within 272.5–275.0 nm. The polydispersed particles had sizes ranging from 25.00 to 191.41 nm. The antibacterial and antifungal activities of the TiO_2_ NPs were remarkable, while they also displayed good dye degradation, DPPH and hydrogen peroxide scavenging activities as well as prevention of coagulation of human blood *in vitro*. This work represents the first report on the use of extracts of different parts of *C. nitida* for the synthesis of TiO_2_ NPs, and for both hydrogen peroxide scavenging and anticoagulant activities. These sets of properties of the synthesized TiO_2_ NPs would impact positively on their exploitation in healthcare and environmental applications.

## Declarations

### Author contribution statement

Akinola, P.O.: Performed the experiments; Analyzed and interpreted the data; Wrote the paper.

Lateef, A.: Conceived and designed the experiments; Analyzed and interpreted the data; Contributed reagents, materials, analysis tools or data; Wrote the paper.

Asafa, T.B., Irshad, H.M.: Analyzed and interpreted the data.

Beukes, L.S., Hakeem, A.S.: Analyzed and interpreted the data; Contributed reagents, materials, analysis tools or data.

### Funding statement

This research did not receive any specific grant from funding agencies in the public, commercial, or not-for-profit sectors.

### Competing interest statement

The authors declare no conflict of interest.

### Additional information

No additional information is available for this paper.
